# Key stakeholders’ experiences and expectations of the care system for individuals affected by borderline personality disorder: An interpretative phenomenological analysis towards co-production of care

**DOI:** 10.1371/journal.pone.0274197

**Published:** 2022-09-22

**Authors:** Laura Friesen, Graham Gaine, Ellen Klaver, Lisa Burback, Vincent Agyapong

**Affiliations:** 1 Department of Educational Psychology, University of Alberta, Edmonton, Alberta, Canada; 2 Addiction and Mental Health, Alberta Health Services, Edmonton, Alberta, Canada; 3 Department of Psychiatry, Faculty of Medicine, University of Alberta, Edmonton, Alberta, Canada; Universita degli Studi di Milano-Bicocca, ITALY

## Abstract

**Background:**

The diagnosis of borderline personality disorder (BPD) consists of extreme emotional dysregulation and long-term disability when left untreated. It is associated with ineffective use of health care systems and mismanaged care in emergency departments, which can result in a revolving door phenomenon of urgent system usage, poor treatment outcomes, or patients falling out of care entirely–all of which primarily affect patients with BPD as well as their caregivers and clinicians. This crisis must be addressed with a comprehensive understanding of key stakeholder perspectives on the challenges of the system and potential solutions.

**Objective:**

This study explored the perspectives of three key stakeholder groups (i.e., patients, clinicians, and caregivers) in relation to their experiences with and future expectations of the care system for those affected by BPD.

**Methods:**

Four patients with BPD, three generalist clinicians with experience treating BPD, and three caregivers of individuals with BPD participated in individual semi-structured interviews. Participants were asked about their experiences with the current healthcare system and their suggestions for improvement. Responses were analyzed using interpretative phenomenological analysis.

**Findings:**

In-depth analysis of the qualitative data revealed twelve shared themes and three themes that were unique to each key stakeholder group. These themes are discussed and used to inform recommendations for promising practices, policies, and training in this area.

**Conclusion:**

Findings support the importance of a comprehensive mental health system approach for improving the accessibility, effectiveness, and acceptability of the management and treatment of BPD.

## Introduction

Borderline personality disorder (BPD) is a condition characterized by extreme emotion dysregulation that is often compounded by other mental and physical comorbidities. The disorder permeates all aspects of life and, left untreated, individuals with BPD are at greater risk of unemployment, work-disability, and suicide [1]. Several characteristics of BPD make this disorder notoriously difficult to treat, including difficulties committing to and engaging in treatment, and establishing a trusting collaborative relationship [2]. Further, BPD is often misunderstood, poorly treated, and stigmatized within healthcare systems [3]. Specialized evidence-based treatments have been developed for individuals with BPD [4–6] but they require such extensive clinician training and financial support that the supply of these intensive psychotherapies may never meet the growing demand [7].

In lieu of specialist psychotherapies, individuals with BPD often receive generalist services and pharmacotherapy, despite limited evidence supporting the effectiveness of medication in this population [8, 9]. Alternatively, patients are placed on waitlists for specialized services, but these lists are typically long, extending suffering and increasing the risk of functional decline [10]. Waitlisting patients also puts tremendous pressure on caregivers who support loved ones in need often without receiving any psychoeducation or skills training for managing BPD [11, 12]. Without specialized care, individuals with BPD are forced to rely on family physicians [13] and urgent care services such as emergency departments and distress lines [14]. Like caregivers, non-specialist professionals and volunteers are often ill-equipped or unprepared to work with this population [14, 15].

Patients, caregivers, and clinicians are key stakeholders in the care system for BPD. Their lived experiences in the care system offer invaluable insight into the depth of the crisis. A recent meta-synthesis of qualitative research highlights patients’ experience with neglectful treatment, including extreme prejudice and stigmatization, involuntary hospital admission, and poor discharge planning [16]. It is also well-documented that patients with BPD often perceive professionals to be unwilling or uninterested in becoming involved in their treatment [6]. Understandably, patients feel that care and treatment will continue to be unsatisfactory [17] and unendingly difficult [18]. These qualitative results are corroborated by quantitative data that show, for example, nearly all survey-respondents with BPD perceived significant unmet needs in their care [19].

Unfortunately, some mental health providers report negative attitudes toward individuals with BPD and, as a result, show less empathy to this population [20, 21]. Healthcare providers’ stigmatization of BPD may partly stem from their own feeling of being unable to treat this population and inadequacies of the healthcare system [22]. The dearth of training and education contributes to clinicians’ negative attitudes toward this difficult-to-treat population [20, 23]. For example, a lack of specialized training often leads generalists to emotionally distance themselves from individuals with BPD [3], which can be especially detrimental for people living with BPD where interpersonal triggers [24] and therapeutic alliance [18, 25] are believed to play an especially crucial role in the course of treatment [26, 27].

The duress experienced when working with patients with BPD may explain why healthcare staff are often relieved to know that a patient has a caregiver to provide support outside of the healthcare setting [28]. Accordingly, caregivers are burdened with the sole care of the patient and often feel their loved ones are more likely to be ignored if the provider is aware that the caregiver is available [29–31]. In addition to feeling ill-equipped to support their loved one, caregivers also feel left out of the treatment process [30–32]. Even when they play an active role in the patient’s life, caregivers feel ultimately overlooked in the treatment process [11, 33]. Together, these findings depict the widespread negative impact that the current care system has on those most closely affected by BPD; leaving patients to fall through the cracks, clinicians to be ineffective and frustrated, and caregivers to feel alone and overwhelmed.

Fortunately, there are potential solutions that are emerging in the literature. For example, a stepped care model [34–36] follows the principle of offering short-term interventions as a first step, even if the disorder has been present for a long time. Long-term, resource-intensive treatments are then reserved for patients who do not benefit from these first-line short-term interventions. Within a stepped care system, long-term therapy is required for a minority, thereby freeing up access to treatment for the majority, shortening (or eradicating) waitlists, improving outcomes, and limiting reliance on emergency departments [36]. Another option to improve the care system for individuals affected by BPD is Good Psychiatric Management (GPM) [37], where non-specialist providers are trained in Structured Case Management (SCM) and basic interpersonal skills for managing the complexity of BPD [23]. Stepped care and GPM hold great potential in repairing and renewing the care system for individuals affected by BPD; however, these strategies often rely heavily on system-wide changes that typically take extended periods of time to achieve.

One promising avenue for designing a healthcare system that is more responsive to the complex needs of those with BPD is co-production [38–40]. As it relates to healthcare, co-production is where “professional services are designed, developed and/or delivered *with* or *by* people instead of *for* them” to secure positive health outcomes [41]. Co-production is built on collaboration of multiple stakeholders with a shared goal of developing a strengths-based, consumer-driven system.

Existing studies investigating the experiences of patients, caregivers, and clinicians affected by BPD have primarily focused on one key stakeholder group at a time. Previous inquiries have also been limited by a focus on negative experiences with the current care system. While it is helpful to identify problems to be addressed, effective co-production also requires an understanding of what is already working and potential solutions [see 42, for review]. Accordingly, our research team sought to explore and compare key stakeholders’ negative and positive healthcare experiences, along with their perspectives on how the system can be improved.

## Method

Qualitative interpretative phenomenological analysis methodology [43] was used to explore the experiences of having BPD or supporting an individual with BPD, and to learn about stakeholder perspectives of what is needed to fill gaps in current healthcare systems. For a detailed account of the methods, procedures, and evidentiary support for the soundness of this in-depth study, the reader is directed to the published research protocol: https://www.researchprotocols.org/2020/8/e14885 [PROTOCOL DOI] [44]. The current study received ethical clearance from the University of Alberta’s Health Ethics Research Board (Ref. # Pro00086416). A brief summary of the methodology follows.

### Design

IPA is an exploratory research methodology that uses semi-structured interviews and broad open-ended questions to gain a more sophisticated understanding of the topic of interest [43]. Qualitative methodologies aim to provide insight into what it is like for those with first or second-hand experiences to help to answer “why” questions and provide rich, vivid descriptions of these experiences [45]. Exploratory research also serves to give insight into the experiences, needs, values, motivations, and preoccupations of stakeholders, which contribute to the development of effective and economically-sound programs and services [46]. IPA effectively explores phenomena with small sample sizes and in-depth, rich interviews. IPA was chosen given that the aim of this research was to better understand the unique individual experiences, needs, and perspectives of key stakeholder groups. IPA explores phenomena starting with idiographic experiences, and then allows for aggregation of the data into larger themes. The data analysis began with idiographic analysis of each individual interview, followed by within-group analysis, and then finally across-group analysis. The focus of this paper is primarily on the across-group analysis findings. To help participants prepare for the interviews, participants completed pre-interview activities (PIAs) in the week prior to their interviews. PIAs have been found to supplement the interviews, and can include diagrams, lists, or drawings that are completed in response to guiding questions informed by the research questions [47]. The benefits of incorporating pre-interview activities include triggering memories and encouraging active reflection of the topic in more depth. The interviews began with a discussion of the completed PIAs, and then moved into the semi-structured interview protocol.

### Participants

This study took place in Edmonton, Alberta, Canada within the province’s publicly funded healthcare system within the Addiction and Mental Health program. Individuals with a diagnosis of BPD, caregivers of individuals with BPD (i.e., family, partners, closely involved friends), and clinicians with experience supporting and treating individuals with BPD (i.e., mental health therapists) were invited to participate in the study. A total of 10 participants were recruited: four patients, three caregivers (all mothers), and three clinicians (one psychiatric nurse and two social workers) agreed to share their experiences. None of the participants were in dyads or triads with each other (i.e., no caregiver-child-therapist relationships existed amongst participants to the researchers’ knowledge). All participants were 18 years or older and female. All participants provided verbal and written informed consent to participate in the study. A sample of 10 participants is in line with IPA research guidelines [43]. Pseudonyms are used to protect the confidentiality and privacy of the participants.

### Data collection and analysis

Interviews ranged from one to two hours and data were analyzed according to the guidelines developed by Smith and colleagues [43]. The interviews resulted in an enormous amount of rich data. The data was primarily analyzed by the first author who also conducted all the interviews. Analyses followed Smith and colleague’s six steps including 1) reading and re-reading, 2) coding, 3) clustering, 4) iteration, 5) narration, and 6) contextualization [43]. Following extensive analyses, starting with ideographic analyses which were then followed by within, then across-group analyses, the findings were evaluated carefully and rigorously with the support and review of the research team. Evaluative criteria were followed to increase the findings’ trustworthiness, comprehensiveness, usefulness, persuasiveness, and coherence [48, 49], a process that is detailed in the published research protocol [44]. Examples of verification methods used to enhance the goodness of the qualitative research included member-checking in the interview and audits by and with the research team.

An important process in the hermeneutic circle is that of researcher reflexivity. The first author offers a statement in this section to illustrate the process of this co-constructed research and to locate herself within the research: “*Researchers come from different histories and contexts and hold identities*, *values*, *motivations*, *and preoccupations that inevitably impact the ways in which they make sense of phenomena*. *Participants also have unique histories*, *contexts*, *and meaning-making systems that impact the way in which they experience their lives*. *The PIAs and semi-structured interviews help the researcher understand more about the individual*, *their context*, *and what matters to them*. *Now*, *I locate myself as a researcher*, *a registered provisional psychologist*, *a graduate student*, *a woman*, *and a rural individual*. *These are but a few of my various identities*. *As a rural woman*, *currently located in an urban setting*, *much of my research interests lie in inequity of services and vulnerable populations*. *I am interested in the uncovering of truths (i*.*e*., *the concept of Alethia)*, *specifically those truths that appear to have been misunderstood*. *While I sat face-to-face with the participants in this study*, *I felt I was bearing witness to truths that needed to be better understood*. *As we (the participants and I) talked together about the topic at hand*, *I felt that we were starting to develop a common language*. *As I worked through the initial individual analyses*, *I felt the language begin to refine and over time*, *by returning again and again to the data*, *clear stories begin to emerge*. *Following multiple interactions with the stories*, *I brought what I found to the team and together*, *we worked to refine the common language again*. *We reflected and mused and consulted until we were all fluent in this new beautiful*, *rich*, *storied language that the participants themselves taught us*. *It is the hope of this research team that the findings presented here will express the truths told by the 10 participants in a language that everyone can understand” ~ Laura Friesen*.

## Findings

**Fig 1:**
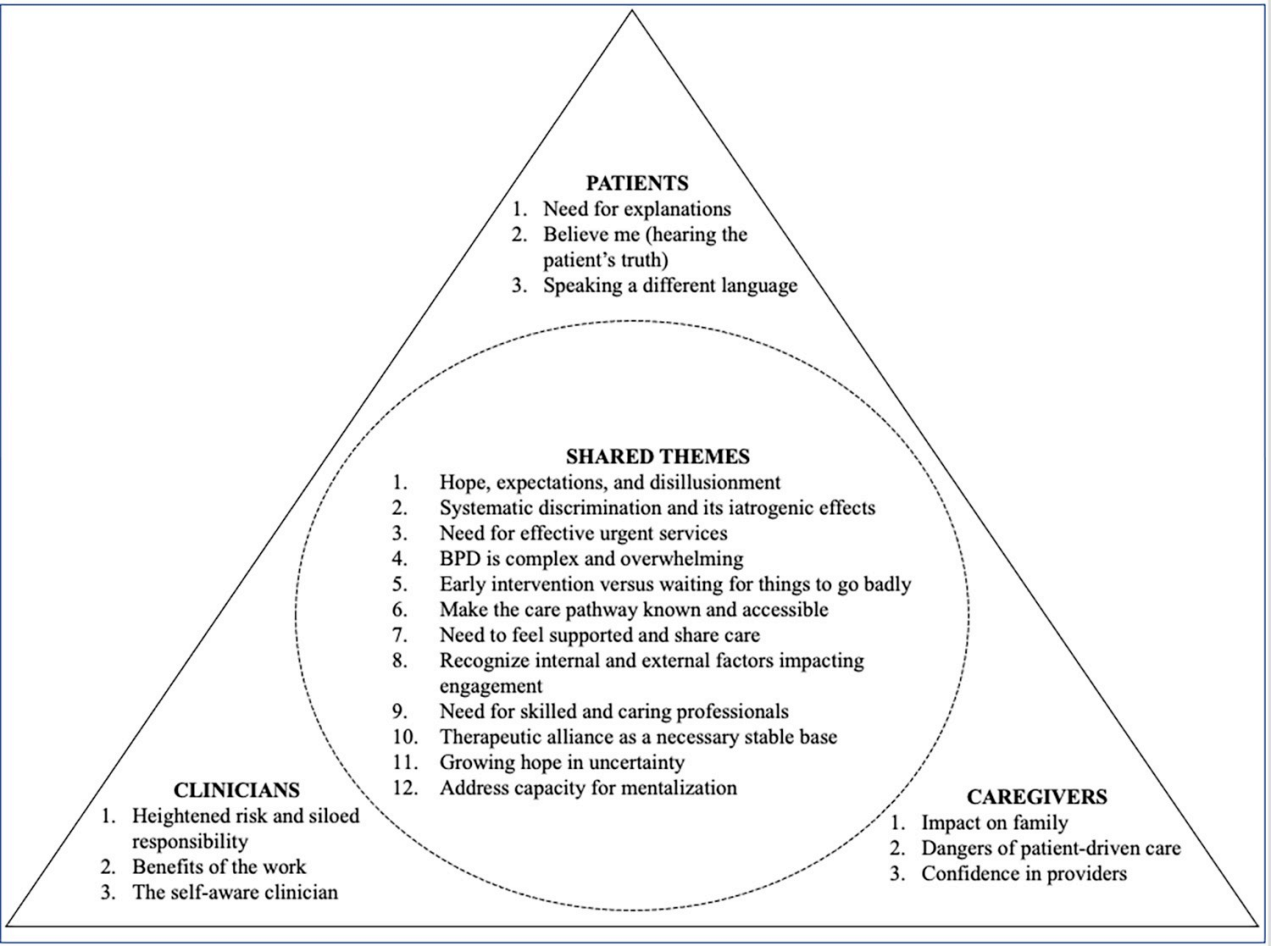
Summary of shared and unique themes of different stakeholder groups.

The in-depth qualitative analysis resulted in shared and unique themes. There were twelve shared themes and three unique themes for each of the three key stakeholder groups–each of which are described below, summarized in Fig 1, and supported by participant quotes. Some of the quotes are in the prose and additional supportive quotes can be found in Tables 1–4.

**Table 1:** Key stakeholders’ shared experiences with and expectations of the healthcare system.

Theme	Representative quotes
**1. Hope, expectations, and disillusionment**	*“I expected after her diagnosis*, *they would say “okay well this is how we fix this*. *Here’s some therapy for her*, *here’s her group therapy*, *here’s some stuff” instead of saying there’s nothing for her*.*” (Caregiver)* *“People with a high level of risk should receive services in a succinct manner*. *We all know that wait lists are a huge barrier*, *the cost of having specialized programs are a huge barrier*, *competency is a huge issue*, *and people that have high acuity*, *need competent individuals*. *So I think that while these are known factors*, *barriers… Being able to help people to access services*, *when they need it in the moment*, *I don’t know that anybody has figured that out yet*.*” (Clinician)*
**2. Systematic discrimination and its iatrogenic effects**	*“All they saw was this horrible human… And I’m like*, *“but I’m not that person*. *I’ve never been that person*. *This is what I meant*.*” And they’re like*, *“no because this is what you did*.*” I’m like*, *“but*.* *.* *. *ugh…” So that’s really what BPD’s like is*, *my path is here and the world sees me doing that… it’s frustrating*.*” (Patient)* *“We need to discern*, *not judge… I very much think about how healthcare can exclude people and oppress people and invalidate and contribute to chronic invalidation*. *And stigma and judgement and othering*.*” (Clinician)*
**3. Need for effective urgent services**	*“I just find like it’s a huge waste of time*. *I don’t mind sitting in the ER for two days straight if something’s going to come out of it*. *I don’t care*. *I will stay there… but when it’s just for nothing and she knows it’s nothing*.*” (Caregiver)* *“It’s also well-documented that the excuse for that behavior is that we have a very fast-paced healthcare environment*. *I’m not going to deny that we are all working very hard*. *But I just don’t think it excuses the behavior*. *And I don’t think that it should be tolerated*.*” (Clinician)*
**4. BPD is complex and overwhelming**	*“It’s misunderstood by so many people*. *Those that work with it*, *those that live with it*, *those that support us*.*” (Patient)*
**5. Early intervention versus waiting for things to go badly.**	*“I need some place for… early education for emotional dysfunctional kids*. *If you do not want to diagnose them early on*, *give [caregivers]*.* *.* *. *because I think… perhaps*, *if we had the tools sooner*, *it wouldn’t get to where we are now*.*” (Caregiver)* *“She’s got these scars that she’s going to live with for the rest of her life*. *How nice would it be to be able to catch that before that happens*? *I guess they wouldn’t be red flags*, *they’d be little*, *maybe pink flags*.*” (Caregiver)*
**6. Make the care pathway known and accessible**	*“I know that there’s probably other things out there too*, *but I don’t know about them…It’s just frustrating to know there’s this thing that can help*, *[but] you can’t get it*.*” (Caregiver)* *“There’s such a long wait list*. *It takes two years to get in*! *So for people like this client… they can’t wait two years*! *They’re going to be dead in two years*! *So we’re just trying to*, *you know*, *keep her bandaged up until then… that’s a system problem*. *So we know*, *we know what she needs is full-on DBT but there’s none available*.*” (Clinician)*
**7. Need to feel supported and share care**	*“*.* *.* *.*It’s my family and friends that [are] very conditional and my care team is unconditional*. *They are with me 100% the way*. *I’m very lucky… I have this feeling that*.. *that… I am [one of] the lucky few that do get treatment*.*” (Patient)* *“Individuals who present at multiple points of care are trying to give us information just as they’re trying to manage the information that their system and their body is giving them… There’s no malice behind showing up at multiple points of care*. *They’re genuinely trying to get their needs met*. *This is an individual who’s suffering and sometimes I think we have to use an anti-stigma strategy*, *where you use empathy or phenomenological empathy to be able to support [patients] in the way in which [they’re] viewing information*. *We need to discern*, *not judge… I very much think about how healthcare can exclude people and oppress people and invalidate and contribute to chronic invalidation*. *And stigma and judgement and othering*.*” (Clinician) *
**8. Recognizing internal and external factors**	*“The only reason I have made permanent capable changes in my life*. *Because he followed*, *he allowed my pace to happen… I think that’s what’s made the difference*, *is allowing me to dictate the pace*. *And allowing me the time I need to make these changes permanent*. *I’m going to flounder*. *I’m going to fail*. *I can accept that now*. *But I have the tools to pick it back up and keep walking forward*. *Because I’ve had this experience and this time*.*” (Patient)*
**9. Need for skilled and caring professionals**	*“I wish there was more time with whoever I was talking to… someone who knows what they’re doing… It felt like a lot of therapists I’ve met just wanted to do therapy but not have like a specific route of therapy*.*” (Patient)* *“…it’s good to have [resources] but not if*.* *.* *. *unless they’re going to be effective*. *Like spend the money but spend it wisely*.*” (Caregiver)*
**10. Therapeutic alliance as a necessary stable base**	*“[Clinic] is my foundation*, *this is my safe zone*. *…Foundations are massive for me… because I cannot go anywhere if I don’t have somewhere to start…” (Patient)* *“We really have to work very hard to have a trusting relationship*. *That’s the least we can do*. *And if you’re in this profession*, *you should care*. *As a bias*. *And I know I use the word should*.* *.* *. *but you should care*. *You shouldn’t be in a helping profession if you don’t*. *And people go through a lot*.*” (Clinician)* *“I think we have to use an anti-stigma strategy*, *where you use empathy or phenomenological empathy to be able to support [patients] in the way in which [they’re] viewing information*.*” (Clinician)*
**11. Growing Hope in Uncertainty**	*“I began to be disillusioned that the mentally ill person was in charge of all the decisions for the care when she’s so sick… they don’t have the sight to sort out their own physical sensations*, *the observations*, *the judgements and what’s in their best care*. *And a lot of them don’t even want to be alive*. *That’s their coping*: *I just want to disappear and die*. *So how can we put someone who is saying*, *I don’t want to live because all I experience is pain*. *How can we put them in charge*, *to say*, *you get to make the best decisions about your care*? *Because my daughter has actually come out and said*, *there maybe are things that would help*, *and it would just prolong my pain*, *and so I don’t want to do them*, *because I don’t have faith enough that they’ll make me all the way better*, *but it’ll just prolong my living*, *so why would I do those things*?*” (Caregiver)*
**12. Address capacity for mentalization**	*“They have to deal with a lot*. *There’s a lot of sick people out there… they are trying*. *They are doing the best they can*.*” (Patient)* *“My [daughter]… was seeing her oldest sibling just cycling around and [said] “I’m upset that she’s not making the changes that she needs*.*” …I was able to help her understand through a neat analogy*. *She was cold in her room and we turned on the heater*.* *.* *.*and I say*, *you’re cold*, *and you have a way to warm up… but imagine if you didn’t*. *imagine if you’re just in the cold*, *in the dark*, *and you had no resources*. *And she just started to cry*, *and I said “that’s how your sister feels*. *She’s in a dark cold place and she doesn’t have the skills and I wish that she did*.* *.* *., *but she doesn’t*.. *I know it’s a sacrifice*, *but we can’t leave her there alone*, *we need to walk this journey with her*.*” (Caregiver)* *“Individuals who present at multiple points of care are trying to give us information just as they’re trying to manage the information that their system and their body is giving them… There’s no malice behind showing up at multiple points of care*. *They’re genuinely trying to get their needs met*.*” (Clinician)*

**Table 2:** Patients’ unique experiences with, and expectations of, healthcare system.

Theme	Representative quotes
**1. Need for explanations**	*“When I came here to see [psychiatrist]*, *I told him like*, *I’m cutting myself*, *I’m doing this*, *I’m doing this*. *And he knew exactly what to do and what to say to me… he defined the disorder*.* *.* *. *He listened to me*. *He understood what I was trying to say*. *He would ask and clarify things for me*. *He always booked a follow-up… he doesn’t make me feel like crap for being on meds*.*”* *“So when I’m incapable of doing something they can do with breeze*, *they’re like what the [explicit] is wrong with you*. *Why aren’t you getting this*? *And it’s like*, *“because my brain can’t do that*!*” And they’re like*, *“how could your brain not do that*?*”[…*.*] We’re the same end of the bridge*. *Just looking two different directions*.*”* *“It’s… the different ways it’s been presented*. *When the lightbulb goes on it’s like…*. *Literally everything just goes click and… now I understand why*. *Now I understand how*. *Now I can work on fixing that part of it*. *…I find that when I do get the lightbulb the confusion does sort itself*. *But there’s still things that I just can’t- like de-escalation*? *I know what that means*. *I have no concept of what it is*.*” *
**2. Believe me (hearing the patient’s truth)**	**“***I’d even say to [psychiatrist]*.* *.* *., *“you’re not helping me*, *you’re not helping me*.*” And I know people with BPD sometimes can be very persistent and angry because of their symptoms and that was me*. *She would just take it as me being rude or angry and I’m just like*, *“I’m suffering so bad*,*” like I just didn’t know how to interpret it… it took me to try and kill myself for me to get help*. *…I think that when they hear someone saying*, *“I need help or I need this…” They’re just taking it kind of lightly*. *I know like a lot of people do take mental health… seriously but I just feel like when someone’s like I really need this*, *or like I don’t feel safe or I’m scared or I don’t know what’s going on with me*, *… they could be more welcoming*.*” *
**3. Speaking a different language**	*“It’s really hard to explain that to somebody how I feel*. *No one understands unless they have it… [Caregivers] need education and they need to know what it’s like*. *They just need to be aware of my triggers and they need to know how to talk to me*.*” *

**Table 3:** Caregivers’ unique experiences with, and expectations of, healthcare system.

Theme	Representative quotes
**1. Impact on family members**	*“It’s been a journey that at times I have felt like oh my goodness*, *I feel so depressed because*.* *.* *. *how can you not*. *When someone you absolutely love and adore*, *and you’re seeing them not even want to live… It’s finding grief in little things*.*”*
**2. Dangers of patient-driven care**	*“It’s not that we can mandate certain things*, *because she needs to be involved… family and loved ones [also] want to be respected and listened to and included…”*
**3. Confidence in providers**	*“…all of a sudden I find myself in a room with a doctor and three psychiatric nurses standing there trying to tell her to stop scratching*. *And I’m thinking oh my goodness*. *“Somebody please get me an ice cube right now*. *Get me an ice cube*.*” So they’re like*, *ok… run and get an ice cube*. *I’m putting an ice cube in my daughter’s hand and holding it*. *Going*, *why are they not doing these skills*? *Why are they not putting cold water*, *a cloth*, *why are they not tipping the temperature kind of thing*? *Like what’s going on*? *Why do I know this from reading on my drive*, *more skills*, *than what they’re doing*.*”*

**Table 4:** Clinicians’ unique experiences with, and expectations of, healthcare system.

Theme	Representative quotes
**1. Taking responsibility**	*“I don’t want people like that on my caseload… I can’t see her every week… and we don’t have any program to provide the support she needs*.* *.* *. *but she’s on my caseload*. *So if she dies… then … guess whose door they’re gonna come to*? *Mine*.*”* *“There’s a lot of trying to*.* *.* *. *transfer people out or in to us or whatever just because people don’t want to work with some of these people*.*”*
**2. Benefits of the work**	*“[Working with this population has] been the most rewarding part of my career*..* *.* *.*I’ve worked for a really long time*. *But I think since having the training*, *not only do I feel competent*, *but I feel like… the vulnerability that people show me and are willing to sit in*, *is the biggest gift I’ve been given in my whole career*.*” *
**3. Self-Awareness of the Clinician**	*“*.* *.* *.*Countertransference is a big issue with BPD*.*” *

### Themes of shared experiences and expectations

#### 1. Hope, expectations, and disillusionment

Participants described how generally, they felt hope initially for effective treatment, but this hope was often diminished by the many barriers to obtaining quality care. One patient described her experience:

*After I was diagnosed with BPD, interacting with the healthcare system was like finding cell service in the middle of nowhere. In certain places I could connect right away, have strong support and access. In other places, I could get some support and access but not regularly*.

**Fig 2:**
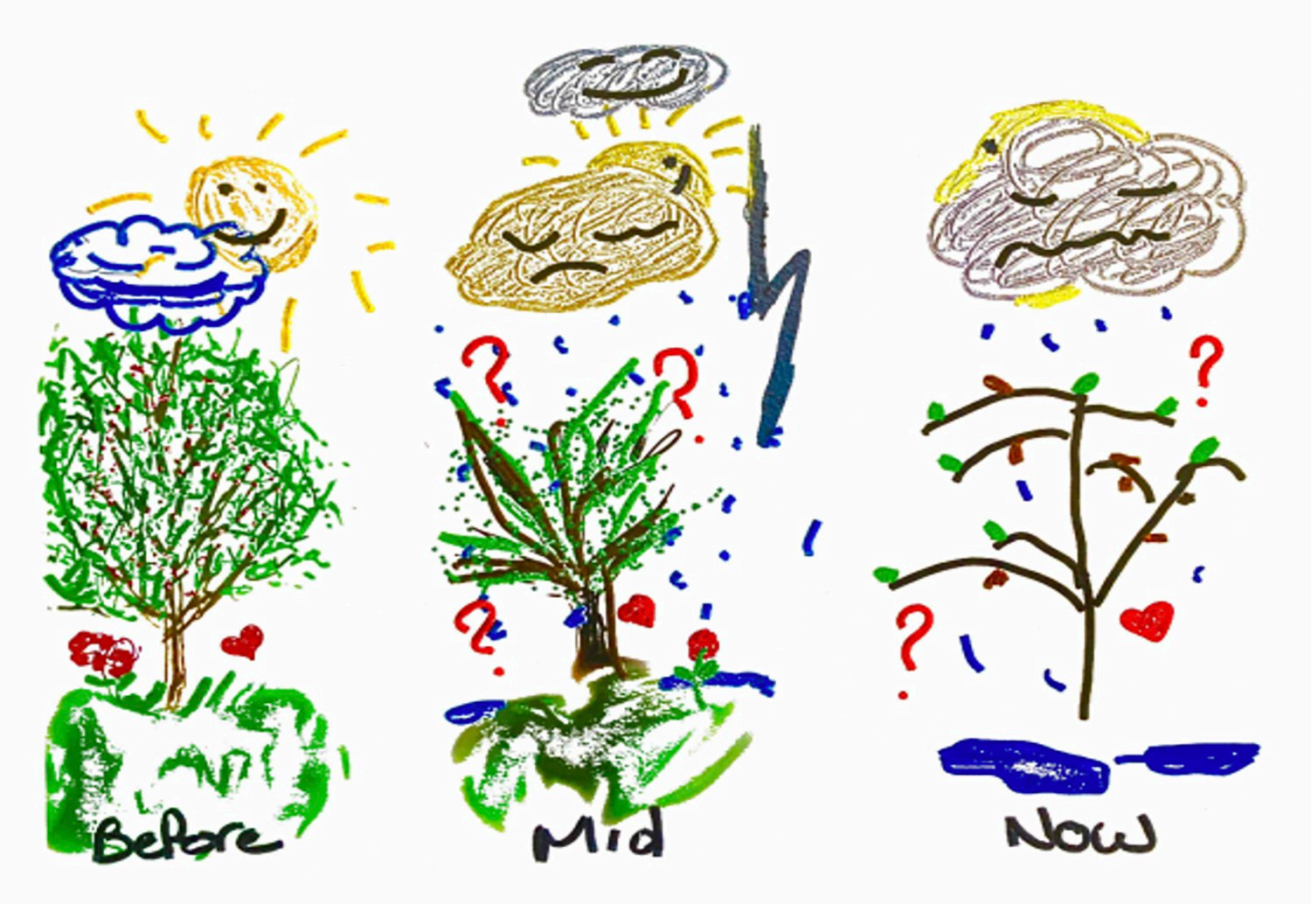
Iris’ drawing of the experience of BPD.

The following pre-interview activity image, shown in Fig 2, was completed by a caregiver (Iris), and illustrates the experience of starting with hope and facing disappointment and sadness:

The patients, caregivers, and clinicians all described that the healthcare system is making attempts at improvement, yet their disillusionment with the system remains strong. Participants referred to ways the healthcare system is attempting to make positive changes for patients with BPD. For patients and caregivers, initial hope was frequently followed by disappointment—for example, believing a diagnosis will lead to treatment options and receiving nothing or inadequate options. Idiographic responses included discussions about efforts to reduce stigma and to increase training, interdisciplinary cooperation, and access (all of which are better represented by separate themes below). What was common to the interviews was the tendency to use tentative language such as, “yes, but,” “there is an effort,” and “attempting.” This suggests that while attempts at system improvement are apparent, the attempts are not yet meeting the needs of this population.

#### 2. Systematic discrimination and its iatrogenic effects

All participants discussed the role of stigma and the need to better understand and humanize the diagnosis of BPD.

*If you show up at the hospital and you’ve got a label of borderline and you’ve hurt yourself and you’ve got lacerations on your forearm… what do you think really happens*? *And I’m just going to be bold and say that I think stigma and healthcare provider bias and/or micro aggression may come into play*. (Clinician)

**Fig 3:**
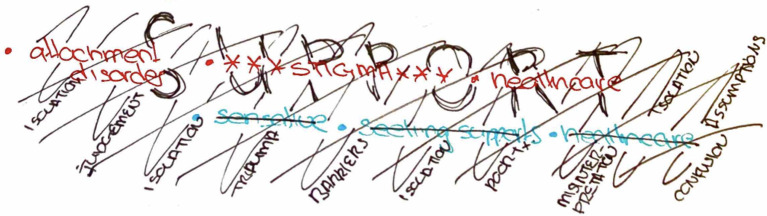
Natalie’s drawing of barriers for BPD patients.

Patients discussed the need for more knowledge and understanding about BPD. Caregivers also identified the negative impacts of stigma and need for increased knowledge. Clinicians identified stigma as a barrier to gaining access to services and discussed the need for system change. Fig 3 below depicts a clinician’s drawing of the barriers associated with systematic discrimination that prevent support for patients with BPD:

#### 3. Need for effective urgent services

In terms of current urgent service options, all participants discussed issues with the overly “medical” focus, stigma, lack of empathy, and perceived judgement when seeking urgent services. While some recognized the strain on emergency departments and lack of time to provide more care to patients, the issues were viewed as a major barrier to treatment. Patients and caregivers placed an emphasis on what it was like to reach out for help in a crisis and face disappointment. Generally, responses included having to wait and then being sent home without treatment, feeling that nothing changes, not being able to talk to anyone, feeling abandoned by the system, and receiving short-term physical treatment (e.g., stitches after self-harm) rather than psychological treatment for the source of the emotional pain. For example, a patient shared her experience:

*I expected a doctor to help me, to be honest. There’s oodles and oodles of times where I’ve been in the hospital with cuts that have to be fixed up and I didn’t get a lot of immediate care there. Well, no I did get immediate care. They fixed the… the cut…*

While several mentioned occasional positive experiences from an empathic practitioner, participants tended to focus on describing areas that stood out as negative or even iatrogenic.

#### 4. BPD is complex and overwhelming

Throughout the interviews, complexity as a theme emerged consistently. The illness itself is complex in that comorbidities are often present, complicating treatment needs and psychological resources available to patients. The type of treatment required can depend on the severity and type of symptoms but also on the capacity or readiness of the patients to engage in various types of treatment. While services may be available, training and the ability to accept patients who present with severe symptoms varies tremendously, often excluding some help-seekers who are then left to “fall through the cracks.” A caregiver shared her overwhelming and complicated experience:

*[She] started cutting, stopped eating, lost crazy amounts of weight, the depression, the anxiety, not being able to keep a friendship with anybody, and so many doctors, therapy, all this stuff, medications. . . And we still had no idea what was going on with her. Trips to the ER because of suicide attempts, no idea. You know, they check her out in the ER, the mental health people come in and talk to you and they send you home with a plan. It was frustrating and so we had the fights and the escalation. . . it didn’t make sense*.

#### 5. Early intervention versus waiting for things to go badly

Participants described how patients often must wait until symptoms and situations have escalated before receiving treatment and how they wished for earlier intervention. Caregivers discussed how they wished they had known what to do when “pink flags” came up in their children. They also expressed the wish that healthcare providers had taken pink flags seriously and offered helpful advice, even if a diagnosis was not yet determined. Likewise, clinicians also described how they wished their patients could be seen earlier before symptoms became more severe and more difficult to treat. A patient’s words describe this succinctly:

*If we have support from day one, education from day one, with the understanding there is nothing bad, you know, nothing that we cannot learn and fix and love. If we can do that… we’re going to save tonnes and tonnes of days and hours and people and time*.

#### 6. Make the care pathway known and accessible

Issues related to lack of resources and access were identified by all participants. Many times, patients, their families, and even clinicians were not aware of services available. Even when services are known, participants described how accessing them was difficult due to problems like siloed care, exclusion criteria, or waitlists. Patients stated they went to emergency departments because they simply did not know where else to go and described wishing there were more accessible options available to them.

*It would have helped if I knew what my resources were*. *Because I didn’t figure them out until a lot later in the journey… all I knew what to do was go to the hospital*. (Patient)

#### 7. Need to feel supported and share care

All participants strongly described the need for different levels and types of support and shared care. A caregiver described a positive experience:

*[The doctor] was validating, she understood my concerns about my daughter going to the [unit]. She heard, took time to hear what my concerns were, and yeah… a few others along the way that also did the same thing. Or encouraged me to get some good self-care, this is going to be a long journey. So just offering their insight and their compassion*.

**Fig 4:**
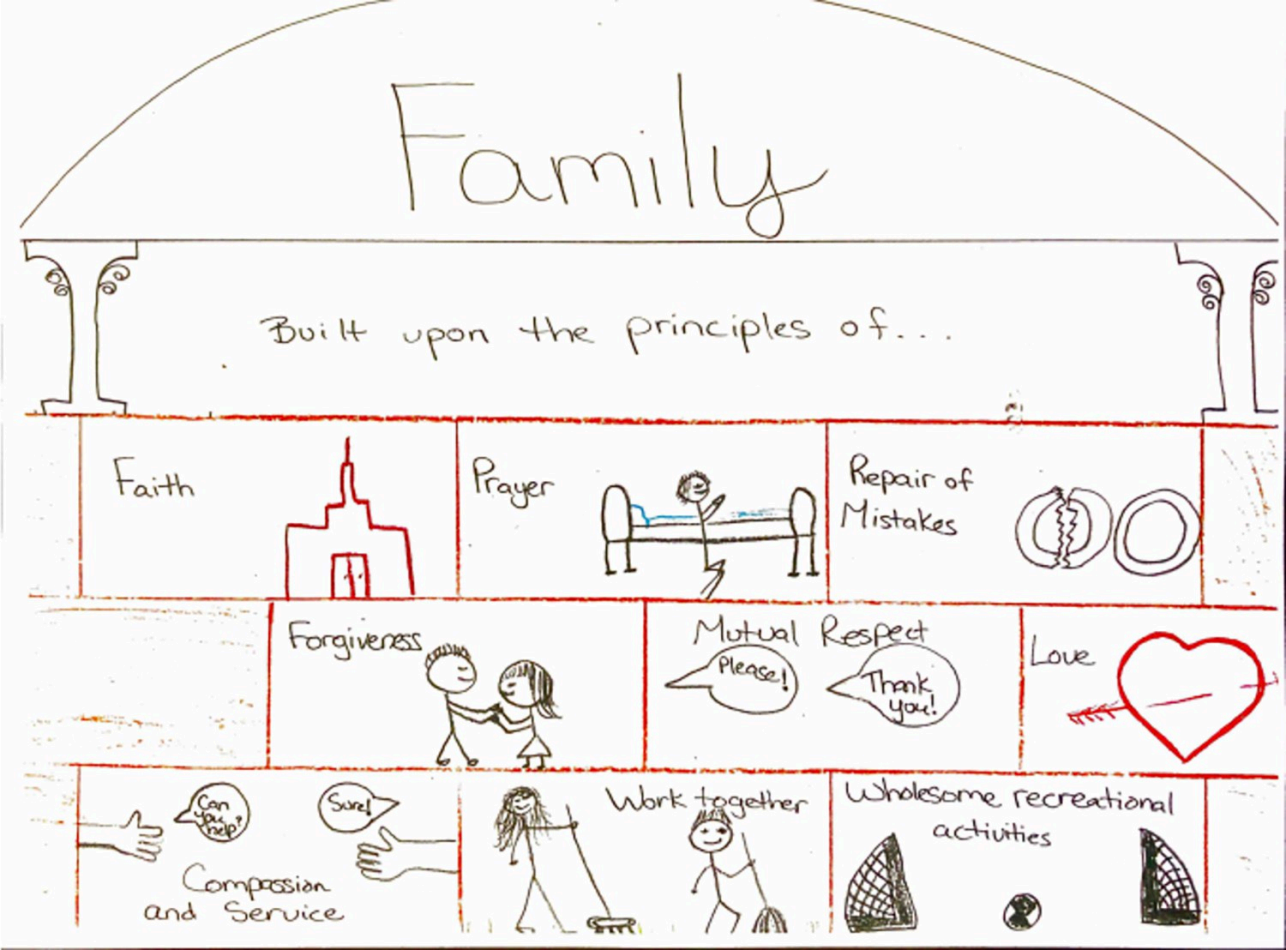
Rebecca’s illustration of the importance of the family system based on her faith organization’s conceptualization.

Clinicians described the need to be supported by the system in terms of training and supervision so that they could better support their patients. Caregivers wanted to be involved so that they could support their family member/patient and they also found it helpful when they were also supported (either by family support groups or by the patient’s clinicians). Participants, especially the caregivers, described the need to include the family in the treatment process since families are often providing support and care for the patients at home. The importance of the family system as support for the patient was especially stressed by caregivers and one mother drew an image, shown in Fig 4, to depict her perspectives of the importance of family:

Shared care may sometimes refer to multiple clinicians caring for a patient. However, it may also refer to different points of contact with professionals who have different roles and responsibilities. Across these different points of care (e.g., police, social worker, emergency department nurses and psychiatrist, family doctor, outpatient therapist, private psychologist etc.), there is a responsibility to be accountable, to include family in care when possible, and to have at least a basic understanding of BPD. While participants acknowledged that symptoms of the illness made effective engagement difficult at times, they also held that patients were responsible to reach out and do their part for treatment to be effective. Finally, clinicians also spoke of the helpfulness of having a team approach. Idiographic references included being supported by colleagues, consulting, working as a team, learning from colleagues, and having supervisors who validate clinicians’ experiences.

#### 8. Recognize internal and external factors impacting engagement

Participants described internal and external factors as impacting treatment engagement. Internal factors included the effects of the symptoms of BPD, acceptance of the diagnosis, and personal motivation to seek and take part in treatment. One patient stated:

*You have to make yourself present for healthcare and want to be well*.

External factors included availability of treatment, waitlists, siloed care, exclusion criteria, clinician characteristics, investment in the treatment of patients with BPD, and situational factors. With complex barriers to engagement including symptom severity and patient readiness, participants described the need for a system with visible services, meet patients at their stage of readiness (wherever that might be), and if needed, hold patients accountable for their part in treatment engagement, while also acknowledging need for system accountability and availability.

#### 9. Need for skilled and caring professionals

Participants voiced their need to match patients with BPD with practitioners who have the required training and skills to effectively provide services. Clinicians in the present study were all generalist mental health therapists, and this was seen as being problematic in the treatment of severe BPD. Specific characteristics that were reported as being important to all stakeholder groups included: specialized training, commitment, providing necessary knowledge and skills with patients, having confidence in providers, insight and self-awareness, and matching need with care. Therefore, while clinician skill and training were seen as necessary, participants also wanted clinicians who are genuinely caring and committed to the well-being of patients. A clinician described the impact on patients when clinicians did not use helpful skills:

*If we’re not using all of these skills as clinicians, transference and/or power hierarchy, medical health system model gets in the way. Contributes to that chronic invalidation and then you have the service consumer floundering in a system of care*.

#### 10. Therapeutic alliance as a necessary stable base

The relational aspects of treatment were emphasized by participants. In addition to building a caring, trusting therapeutic relationship, participants, especially patients, also discussed the therapeutic relationship as a stable base from which patients can do meaningful work. One patient described her experience without a stable base:

*[I was] busy and rotating… and I kept going around between a bunch of people… I just felt alone and it was kind of annoying to be rotating around all the clinicians… I think one thing I did expect was… having the one person I could work with… I just really really needed something solid… it felt like I had to start over every time I saw someone new*.

Specifically, being caring and using basic micro-skills like active listening, validation, and reflections were seen to be vital to the therapeutic relationship.

#### 11. Growing hope in uncertainty

The need for hope throughout the rollercoaster of living with and/or supporting individuals with BPD emerged repeatedly. This theme was typically expressed through participants’ stories of disillusionment and diminished hope from past and/or present experiences. A clinician described her experience of hope and lack of hope when she no longer knew what to do for her patients with severe BPD:

*If they’re more extreme, I don’t know what the hell to do with those people, that is still an open question for me. Get them to a day hospital for three weeks. And hope that, I don’t know, hope that they don’t come back to me, to be quite honest. I hope they don’t come back to me. I can’t do anything for them*.

**Fig 5:**
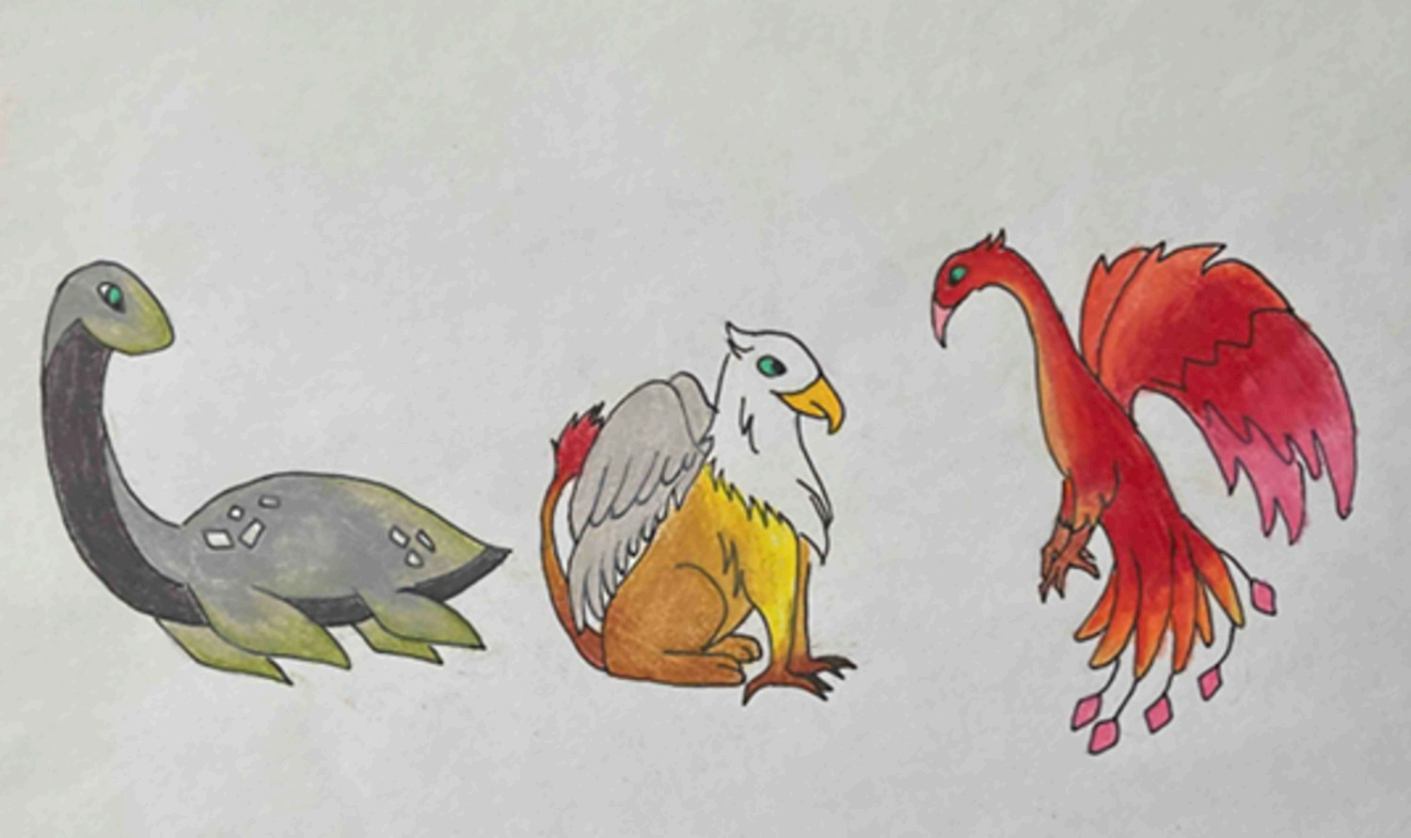
Esme’s drawing of experience of having BPD.

One of the patients in the study drew her BPD experience as a hopeful process shown by mythical creatures who are known for different characteristics and perceptions (see Fig 5).

Rising from the ashes like a phoenix, Esme illustrated how hope can remain or be renewed for individuals with BPD. Hope was said to be installed or reinforced when providers were caring, consistent, competent, and effective. Moreover, having a clear care pathway–somewhere to go for help based on need–would aid in the growth of hope. Even if recovery would take time, to be able to see progress or clear options and to meet professionals who truly wanted to work with patients with BPD, led to increased hope for future recovery.

#### 12. Address capacity for mentalization

The ability to understand the mental state and perspective of oneself and of others is known as mentalization. While patients recognized the limits of staff in emergency services, they expressed a desire for staff to “get [my] brain” and go beyond their appropriate scope of practice, training, and available time in a fast-paced ED environment. Patients also expressed that their experiences could not be understood by people without BPD. Patients also expressed their own difficulty understanding the thoughts and feelings of others. One patient stated:

*I can never be normal. I can never… understand how other people work, think, and understand stuff*.

Caregivers and clinicians expressed an understanding of what it might be like for a patient within the care system. One clinician, taking perspective (thereby mentalizing), stated:

*You know, we look at people’s behaviors and we blame them or we think, you know, we don’t want to work with them or whatever. But if you look back at where this comes from you think, well, you know, and you think of that little kid… Where this person came from, it’s hard not to have empathy*.

### Patients’ unique experiences and expectations

#### 1. Need for explanations

Patients often felt like they did not understand what was happening, thus needing an explanation to better understand the BPD diagnosis and/or treatment rationale and components. A patient shared her experience:

*[I] did not feel comfortable around a lot of it because I went for help and then they’re doing these strange things that aren’t helping and I’m feeling very confused and that makes me uncomfortable. And then it’s like, why did I come here? And I feel like if it’s just more comfortable, it would have been a lot more positive*.

Idiographic perceptions included wanting talk interventions over and above the usual treatment plan of medication, physical stabilization, and observation, followed by a typical discharge and return home.

#### 2. Believe me (hearing the patient’s truth)

Patients described how getting help for emotion dysregulation can be difficult. In seeking help, they want the professional to understand and believe that what they are experiencing is real. One patient described wanting to be heard over and above, addressing the physical injuries:

*There was one time I was in the hospital because I wanted to overdose and then after that they just kind of let me leave. No one talked to me. They just like, unhooked all the IVs and then I walked out*.

#### 3. Speaking a different language

Patients described how their experience with BPD is like speaking a different language. A patient described this experience:

*The biggest issue I have… with someone who doesn’t have BPD, is my translator doesn’t work… And I don’t have a way to help them find a translator. So if there’s more access. . . laymen terms. If there’s just plain English about BPD, more people without it would be able to help those of us with it, work within their world*.

This theme emerged from many stories about the inability to understand–other people understanding patients and the diagnosis, and patients understanding themselves and the treatments they received–and how this contributed to feeling isolated and hopeless.

### Caregivers’ unique experiences and expectations

#### 1. Impact on family

While one of the patients acknowledged that lack of effective preventative care for herself might have a ripple effect phenomenon on her family, caregivers more clearly described what this ripple effect looked like for other family members. Impacts could include grief and loss and less resources going to other children. One caregiver explained:

*My [daughter]… was seeing her oldest sibling just cycling around and not thinking she needed to make any changes. And she said, “mom honestly it just makes me mad, I know I need to be compassionate, but it makes me mad. Because she has pulled resources… away from the rest of the family. . .when you had to leave every night…I was the one… putting my siblings to bed. . . I felt like I had to jump in this emotional role because you were so… involved…it was reasonable, and yet at the same time… I watched it [take] a toll on my younger siblings. . . I’m upset that she’s not making the changes that she needs*.

#### 2. Dangers of patient-driven care

Caregivers recognized the importance of involving patients in treatment planning but also expressed their fears associated with letting their loved one be in complete control of treatment, particularly given the challenges in judgement and impulsivity associated with BPD. A mother described her worries:

*She definitely needs to be included in this. I just think that when the system says, you’re 18 years old, you’re in the driver’s seat, they don’t understand that BPD interferes with their ability to make good decisions and to sort through what the observations are versus what they’ll do…they’ll make judgement calls and interpret wrong. That’s part of what the illness does. So we’re asking them to be responsible for these choices, and doing something that they couldn’t do, you know, when we have patients that [are] blind, we’re not asking them to drive themselves to an appointment. And yet my daughter is in the driver’s seat to her care, knowing that she can’t see clearly, because that’s what the disorder does. …just because they’re 18, does not mean they don’t need those resources of help… The biggest thing is, keep the family in regardless of their birthdate. Don’t put someone in the driver’s seat of their care when they’re not in a position to safely drive*.

#### 3. Confidence in providers

Caregivers described wanting consistent, dependable care from professionals. They wanted professionals who were committed, accountable, and concerned with responsible caring. A mother shared her experience of giving up control as a parent and the weight of having to rely on others:

*You know when your kids are little, you can pick them up and put them where you want them. And you can fix things, you can change stuff for them to make it better. And here you can’t, and I’m literally relying on her primarily. And then the treatment out there secondary. So, it’s completely out of my control*.

### Clinicians’ unique experiences and expectations

#### 1. Heightened risk and siloed responsibility

Clinicians expressed an internal conflict of wanting to help patients at risk but also fearing being blamed if these patients took their own lives. One clinician described:

*Well it’s hard right, because… I’ve checked… fairly often on these clients. . . have they died yet? We don’t know. . . are they back in hospital… I don’t like feeling like it’s my responsibility…*

#### 2. Benefits of the work

While clinicians in this study discussed challenges involved in working with patients with BPD, they also described benefits of the work including feeling what they do is meaningful and being able to see strengths in suffering. They described the value, meaning, and enjoyment derived from their work with clients diagnosed with BPD. The ability to identify patient strengths in the midst of suffering appears to be an important part of meaningful work.

#### 3. The self-aware clinician

Clinicians emphasized the crucial ability to practice self-awareness in the moment with clients and being aware of their own reactions when treating patients. A clinician commented on the impact on and responsibility to respond:

*Sometimes I have found that when I’m with a client who’s “acting out” and behaving, I start to act out as well. It’s like ok, this is not how I want to practice. You know? But you just have to be so strong all the time and learn from your mistakes!*

## Discussion

The present study explored key stakeholders’ lived experiences with and expectations of the care system among those most closely affected by BPD. Individual semi-structured interviews were conducted with four patients with BPD, three caregivers of individuals with BPD, and three generalist clinicians with experience treating BPD. IPA was used to identify themes shared across stakeholders and those that were unique to each group. The following sections offer an interpretation of our findings in the context of the extant literature and our research team’s clinical experience. We offer a series of practice implications that represent a step toward co-production of quality healthcare services for those affected by BPD.

### Current challenges

Riding a rollercoaster is a common analogy that patients with BPD and their caregivers use to describe their experience of living with the illness [50, 51]–the emotional highs and lows are like the many tight turns, steep slopes, and unpredictable changes characterized by a rollercoaster [52]. Our findings show that the rollercoaster has much to do with the current care system for BPD, characterized by complexity and cycles of hope, mismatched expectations, and disillusionment. Patients expected to receive specialized and well-established therapies, such as dialectical behavior therapy (DBT), following diagnosis of BPD, which was also an expectation conveyed by caregivers. Patients and caregivers also expected urgent care service providers to be empathetic and knowledgeable. Meanwhile, clinicians hoped patients and caregivers would understand the limitations of their work, whether it be in urgent services or mental health clinics.

These expectations and hopes may, in part, be fueled by the assumption that structured and well-known therapies, such as DBT, are the only way to treat BPD *and* that these therapies are readily provided and easily transferable in care systems. Instead, like many other studies [7, 8, 18], participants from the present study experienced a lack of effective and timely available services in the health care system. In reality, these specialized, well-established, long-term therapies are, in fact, rarely offered because they require extensive training and resources—both of which most care systems do not have. The discrepancy between what is expected to be offered and what is actually offered is discouraging and leaves individuals to be placed on long waitlists where symptoms often worsen and likely contribute to the widely held misconception that BPD is incurable.

The combination of mismatched expectations for care, increased symptom severity and reliance on emergency care centers contribute to the stigma experienced by those affected by BPD. Other studies report similar findings where lack of options lead patients with BPD to rely on emergency departments, call centres, and other urgent services that are not prepared for or trained to provide therapeutic care [10, 23]. Providers of urgent services are not always neglectful or discriminatory toward patients with BPD. Instead, these professionals are often caring individuals, but providing treatment for BPD may not be in their scope of practice or feasible within the setting. Emergency care is intended to be focused on assessment, triage, and stabilization of acute problems, and therefore, is not a place for long-term psychological intervention. Unfortunately, many services for BPD are limited to attending to acute problems, and not a means of early intervention or specialized treatment.

Participants from the present study described frustration with the care pathway being unknown and that resources, when they exist for BPD treatment, are not clearly communicated. Continuing with the rollercoaster analogy, these results suggest that the current healthcare system options for BPD are not only like riding a rollercoaster, but at times, like riding it blindfolded and with no instructions on what to expect. The complicated nature of the current system, its sometimes invalidating environment, and discrimination can at times, have negative impacts on patients. Iatrogenic effects occur when the care itself causes harmful side effects and further complicates the original disorder. This dilemma poses the question of whether current difficulty in treating BPD is a reflection of the disorder or if it is further complicated by the system itself [23]. The findings suggest that the challenges experienced by patients, caregivers, and clinicians are often a combination of complex disorder-related challenges and hurdles in the system, where barriers to effective treatment (i.e., limits on budgets, time, and resources) result in patients entering a cycle of trying to manage alone, being held on waitlists, and seeking emergency services in crisis situations [53]. Caregivers and clinicians also often feel isolated and insufficiently supported, along for the rollercoaster ride.

### Potential practice implications

**Fig 6:**
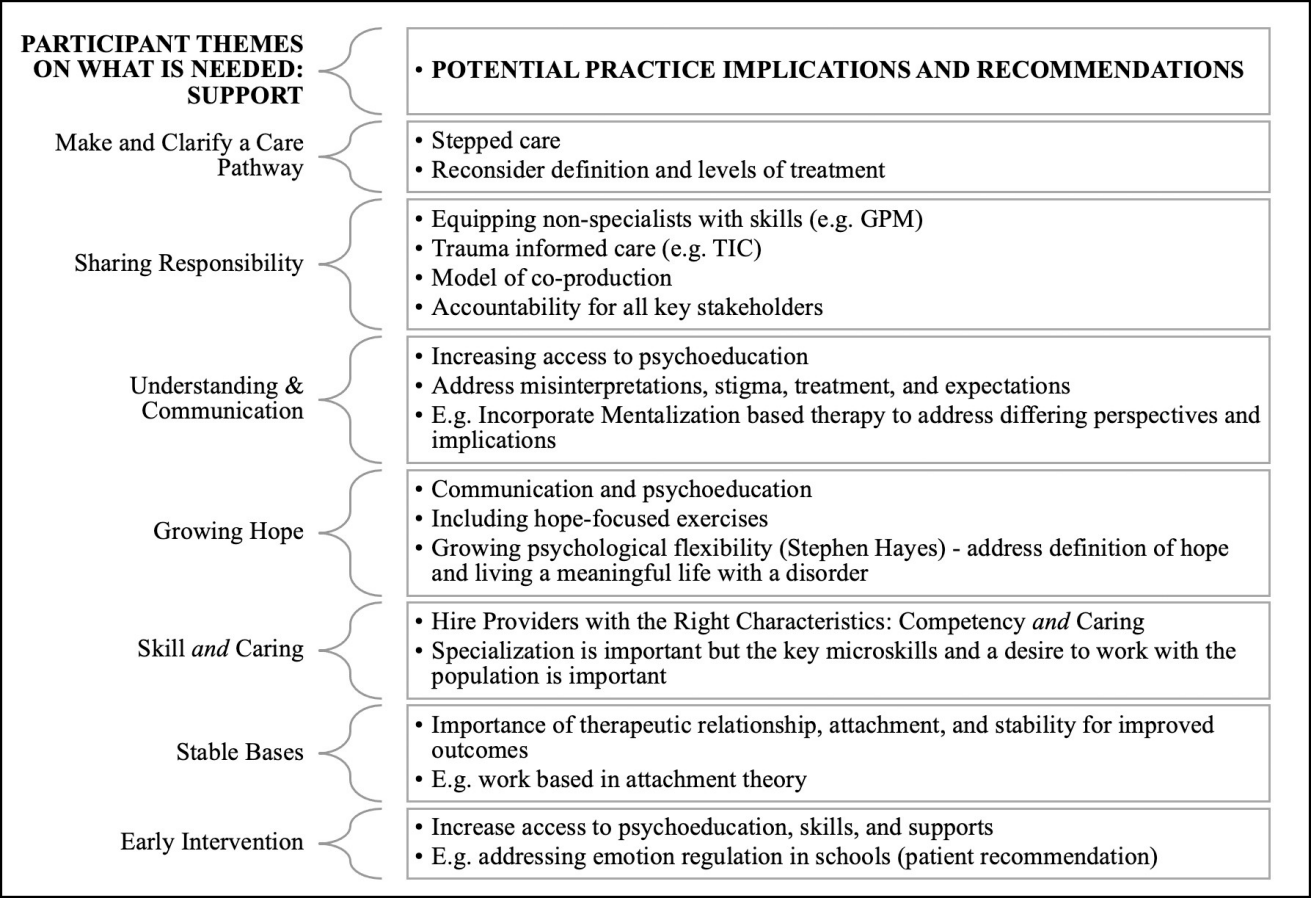
Alignment of findings on what is needed and recommendations.

The first section of the discussion provided an interpretation of the participants’ views of challenges in the current system for patients with BPD, their caregivers, and their clinicians. Below, we outline 10 potential practical implications that align with participants’ responses (see Fig 6 for alignment of findings and recommendations) and may be used to improve the future care system.

#### 1. Adopt a flexible stepped care approach

A stepped care model was described earlier as an emerging option towards improving the care pathway for those affected by BPD. This model can shorten waitlists, aid in reducing the revolving door phenomenon, and use expensive resources and services wisely to better fit budget constraints [36, 54, 55]. Where some patients with BPD benefit from long-term, specialized treatment, other patients do not need this level of care and can benefit from less specialized interventions in shorter term treatment [55, 56]. The staged nature of this model has the potential to address participants’ need for prevention, early intervention, and accessible services [57, 58].

Stepped care was developed to be a flexible approach aimed at adjusting to patient needs [55]. Unfortunately, misapplication of stepped care approaches arise when systems become too inflexible, are unable to respond to changes in patient readiness, or do not incorporate individual needs. Rigid versions of stepped care run the risk that patients end up in siloed care or do not meet strict system eligibility requirements for the service needed; examples of this were described by participants in this study. Stepped care models need to be flexible and take into account client readiness, variable course of the illness, and clinician scope of practice.

#### 2. Reconsider the definition of treatment and recovery of BPD

Our study suggests that redefining treatment and recovery of BPD to reflect more realistic outcomes may clarify misperceptions and expectations among key stakeholders as well as the public. The findings in this study emphasized progress over perfection where there is no “silver bullet”, the importance of treating “pink flags” rather than requiring a diagnosis to provide treatment, and identifying the possibility of effective therapeutic interactions at any point in care. Regarding what constitutes treatment, while not all providers can provide formal psychological treatment, any point of contact can be iatrogenic and thus can also be therapeutic and supportive (e.g., welcoming environments and waiting room interactions with unit clerks, triage or observation in an emergency department with nurses, sessions with psychiatrists, psychologists, or mental health therapists, family doctor appointments, police or social work support, etc.).

#### 3. Equip non-specialist providers

Patients with BPD are frequently supported by non-specialist providers and by those who are not mental health providers (e.g., care aides, group home workers, etc.). Thus, providing non-specialist providers with support and skills is a practice recommendation to improve service delivery at various points of contact. For those providing mental health services, support and skills include ongoing consultation, peer-support, addressing challenges to working in silos, and supervision on the “how to” of interventions and approaches with patients with BPD.

One example of formal training to equip providers is using structured case management (SCM). Good psychiatric management (GPM) is one version of SCM [37, 59]. GPM is a promising approach that holds that the care pathway for BPD can be improved by educating generalist mental health clinicians about the course, core symptoms, vulnerabilities, and response to treatments in BPD populations. The intention of GPM is to reinforce the connection between symptoms and interpersonal stressors as a way to reduce stigma and potentially improve outcomes for BPD [23]. Importantly, care providers at various points of contact, training, and scope of practice can be trained in GPM to improve patient experiences [60].

#### 4. Apply principles of trauma-informed care

Although not explicitly raised by participants in this study, Trauma Informed Care principles may be relevant for addressing participants’ concerns, including stigma, poor engagement with the care system, and the need to feel supported and understood [61]. Research indicates the vast majority of those with BPD have suffered trauma, with a 13-fold greater likelihood of childhood trauma [62], aligning with research identifying trauma as both a risk factor and predictor BPD onset [63, 64]. While trauma is neither necessary nor sufficient for BPD development, the effects of trauma can complicate BPD and its treatment. Worsened emotional dysregulation, altered relationships with others, and the impacts on receptivity to care, interactions with staff, and engagement with services [61] may render standard care models ineffective, worsening the narrative that BPD patients are treatment resistant or “difficult”.

Trauma Informed Care (TIC) is a system wide approach which starts with the paradigm shift from thinking ‘What is wrong with you?’ to ‘What happened to you?’ [65]. Through this lens, many symptoms and behaviors are seen as trauma responses or behaviors that were originally adaptations to adversity. Basic principles of TIC include seeing through a trauma lens, trustworthiness and transparency, collaboration and mutuality, fostering empowerment, choice and control, safety (both physical and emotional), and preventing trauma in the mental health system [65]. For example, in the context of BPD, emotion dysregulation and associated fight/flight/freeze or help-seeking responses are conceptualized as threat responses. Increased self-harm behavior after a relationship break-up may be viewed as an adaptation originally developed to manage intolerable affect related to overwhelming experiences of abandonment in childhood. Following this paradigm, helping BPD sufferers to understand the function of these behaviors, rather than withdrawing from or punishing them, can reduce shame and open the door to finding more adaptive solutions to distress. Viewing BPD through this lens may help providers to become more phenomenologically empathic towards those suffering BPD, reducing stigma, which was repeatedly identified by study participants as a major barrier to care that reinforced a chronic sense of invalidation and abandonment, and contributed to a sense of hopelessness. TIC’s focus on ensuring emotional and psychological safety and emphasizing patient empowerment as much as possible is especially relevant for BPD, given the high rate of interpersonal trauma. Becoming trauma aware also takes into account the impact of traumatic experiences on significant others (e.g., caregivers), and health care providers (e.g., first responders, emergency department staff, and mental health care providers), and works to support these stakeholders appropriately.

#### 5. Move towards a model of co-production

The current research methodology is grounded in hermeneutic principles and emphasizes that knowledge is co-constructed [43]. Co-production in healthcare is a model that is built on collaboration of multiple perspectives with a shared goal of improving care and empowering its key stakeholders to be agents of change in improving their care system [66, 67]. Co-production empowers key stakeholders to take at least partial control of the service agenda while also assuming responsibility [68]. This model is being increasingly recognized as an essential practice in public services and has been shown to improve quality of service, produce more effective outcomes, and in turn, allow for services to be more cost effective in the long run, and reduce staff burn out [69]. The current study aimed to include patient, caregiver, and clinician perspectives and found that the need for shared understanding, clear communication, and involvement of each of the stakeholder groups were important for collaborative and effective services.

#### 6. Personal and shared accountability

It is important to honour patients’ self-determination, particularly when they feel like they have no control in life. The dilemma of when to make treatment decisions for persons with BPD was a strong theme for caregivers and to some degree, clinicians. Patients also saw the need for personal responsibility in treatment but described their behavior as not “being themselves” when they were dysregulated and making poor decisions. The ethical principles of beneficence and autonomy are often at odds and, at times, fitness to drive treatment decisions can shift from hour to hour.

Given the evidence that persons with BPD often experience impaired judgement and other executive deficits, especially during emotional arousal, and may engage in self-damaging behavior, it is tempting to delegate decisions to a proxy for the best interests of the patient. However, given the common experience of invalidation and interpersonal trauma experienced by patients with BPD, taking over care decisions has many inherent drawbacks including a reduction in motivation for treatment, reinforcing a sense of incapacity or failure, and potential for damaging key relationships. Therefore, it may be helpful to train clinicians and caregivers in evidence-based engagement strategies such as motivational interviewing and decisional balance, paradoxical strategies, empathic limits from schema therapy, and engagement of “wise mind” from DBT [70, 71].

#### 7. Accessible and frequent psycho-education

Participants in the present study described the need for more communication and psychoeducation on the illness as well as the process of treatment. Psychoeducation can serve many purposes. Accurate and clear communication with patients and caregivers may reduce misunderstandings or mismatched expectations. Grenyer et al. (2019) provide evidence-based information for clinicians looking to prepare psychoeducation on prognosis and recovery for patients and caregivers [72]. Psychoeducation on diagnosis and effective evidence-based treatment methods would also fulfill the need described by patients and caregivers.

#### 8. Build hope

A theme that emerged consistently was the importance of developing and maintaining hope throughout treatment and recovery and implications when hope was reduced. Similarly, Williams and colleagues [73] found that patients with BPD with lower levels of hope tended to drop out of treatment more frequently. In GPM, authors emphasize the hopefulness that the majority of individuals with BPD get better over time [23]. Hope might be created through psychoeducation, communication, or therapeutic interventions.

#### 9. Hire providers with the right characteristics: Competency and caring

Adopting these potential training and practice implications may also require a practice to hire providers with the right characteristics, namely those who demonstrate competency (e.g. training in treatment approaches like dialectical behavior therapy, mentalization-based therapy, transference-based therapy, schema therapy, etc.) and genuine caring for persons with BPD. While many non-specialist providers can provide positive experiences for patients with BPD, the participants in this study, as in other existing research [5–7], described the need for more specialist providers. Given the rate of presentations to emergency settings, hiring trained professionals (e.g., psychiatrists, psychologists, or social workers) in these settings could also provide patients coming into care with suspected or confirmed BPD with psychotherapeutic crisis support (short-term crisis talk therapy, psychoeducation, defining a care pathway, and referrals with follow-up when possible).

#### 10. Importance of the therapeutic relationship, attachment, and stability for improved outcomes

A robust theme in the current study was that of foundations, stable bases, and the need for a strong therapeutic alliance for good therapeutic outcomes. The importance of attachment theory for the understanding and treatment of BPD is further discussed elsewhere [see 74]. Using evidence-based treatment models that are founded in or emphasize attachment theory and focusing on developing a strong therapeutic alliance appear to be key in the treatment of BPD. The opportunity to work with a case manager throughout the course of care for individuals with severe BPD is also essential to providing a stable base.

### Limitations

Several limitations exist in the present study. One limitation to this study is that only female stakeholders came forward to participate in the study and thus we are missing other key voices and experiences (e.g. a father who is a primary caregiver of a patient with BPD, a male partner, a male patient with BPD, gender minorities, etc.). This study intentionally recruited generalist clinicians who may or may not have specialized training in the treatment of BPD; this is both a limitation and a strength. One of the three clinicians in this study had taken advanced training in the treatment of BPD. Given that we were interested in exploring experiences as they happen (rather than as they would ideally happen), we recruited people who might be able to offer insight into how experiences most commonly play out in the real world with our public health care systems and limited budgets to see what can be done within that system.

### Strengths

The strengths of this study include having in-depth, lengthy interviews which resulted in rich data. Exploratory methods work to uncover values, motivations, preoccupations, rationale, and meaning and these details in context can then be used to develop more meaningful, effective, intentional, and economically sound programs and services. This study was designed to inform the co-production of information to benefit *all* stakeholders (i.e., patients, caregivers, clinicians, management, policy-makers, and government) by learning from those most closely impacted by BPD. Further, inviting a variety of stakeholder perspectives was a major strength of this study, especially given the tendency for misinterpretations and “speaking different languages” (as mentioned by a participant in the findings). The stakeholder groups involved often want the same outcome—a healthy individual living a functional and meaningful life. However, the different groups may come to the table with different approaches, as evidenced by the emergence of unique group themes.

### Conclusion

The health system makes a very significant impact on those with BPD and on those caring for them (positive or negative). The current system is trying to make changes but existing research and the participants in this study hold that the system has a long way to go. There are practical approaches based on the findings of this study, supported by existing research, that can be implemented and evaluated to improve outcomes for patients with BPD and support those who care for patients with BPD.

In the spirit of self-aware and reflexive research, the authors hold that the findings, interpretations, and recommendations that have come out of this study are a positive step forward in refining our understanding of how to best support patients with BPD, their caregivers, and their clinicians. The authors of this study offer these findings and recommendations with the hope that they inform positive change in the current systems and promote promising practices.

## References

[1] Chapman J, Jamil RT, Fleisher C. Borderline personality disorder [Internet]. Treasure Island (FL): StatPearls Publishing; 2021 [cited 2021 Nov 11]. Available from: https://www.ncbi.nlm.nih.gov/books/NBK430883/?report=classic 28613633

[2] Barnicot K, Katsakou C, Marougka S, Priebe S. Treatment completion in psychotherapy for borderline personality disorder: A systematic review and meta-analysis. Acta Psychiatrica Scandinavica. 2011;123(5):327–38. doi: 10.1111/j.1600-0447.2010.01652.x 21166785

[3] Aviram RB, Brodsky BS, Stanley B. Borderline personality disorder, stigma, and treatment implications. Harvard Review of Psychiatry. 2006 Sep;14(5):249–56. doi: 10.1080/10673220600975121 16990170

[4] Cristea IA, Gentili C, Cotet CD, Palomba D, Barbui C, Cuijpers P. Efficacy of psychotherapies for borderline personality disorder: A systematic review and meta-analysis. JAMA Psychiatry. 2017;74(4):319–28. doi: 10.1001/jamapsychiatry.2016.4287 28249086

[5] Oud M, Arntz A, Hermens MLM, Verhoef R, Kendall T. Specialized psychotherapies for adults with borderline personality disorder: A systematic review and meta-analysis. Australian and New Zealand Journal of Psychiatry. 2018;52(10):949–61. doi: 10.1177/0004867418791257 30091375 PMC6151959

[6] National Collaborating Centre for Mental Health (UK). Borderline personality disorder: Treatment and management. 2009. 21796831

[7] Choi-Kain LW, Albert EB, Gunderson JG. Evidence-based treatments for borderline personality disorder: Implementation, integration, and stepped care. Harvard Review of Psychiatry. 2016;24(5):342–56. doi: 10.1097/HRP.0000000000000113 27603742

[8] Byrne G, Egan J. A review of the effectiveness and mechanisms of change for three psychological interventions for borderline personality disorder. Clinical Social Work Journal. 2018;46(3):174–86.

[9] Stoffers JM, Lieb K. Pharmacotherapy for borderline personality disorder: Current evidence and recent trends. Current Psychiatry Reports. 2015 Jan 21;17(1):534. doi: 10.1007/s11920-014-0534-0 25413640

[10] Tomko RL, Trull TJ, Wood PK, Sher KJ. Characteristics of borderline personality disorder in a community sample: Comorbidity, treatment utilization, and general functioning. Journal of Personality Disorders. 2014;28(5):734–50. doi: 10.1521/pedi_2012_26_093 25248122 PMC3864176

[11] Acres K, Loughhead M, Procter N. Carer perspectives of people diagnosed with borderline personality disorder: A scoping review of emergency care responses. Australasian Emergency Care [Internet]. 2018;22(1):34–41. Available from: doi: 10.1016/j.auec.2018.12.001 30998870

[12] Bailey RC, Grenyer BFS. Burden and support needs of carers of persons with borderline personality disorder: A systematic review. Harvard Review of Psychiatry. 2013;21(5):248–58. doi: 10.1097/HRP.0b013e3182a75c2c 24651557

[13] Sansone RA, Farukhi S, Wiederman MW. Utilization of primary care physicians in borderline personality. General Hospital Psychiatry [Internet]. 2011;33(4):343–6. Available from: doi: 10.1016/j.genhosppsych.2011.04.006 21762830

[14] Shaikh U, Qamar I, Jafry F, Hassan M, Shagufta S, Odhejo YI, et al. Patients with borderline personality disorder in emergency departments. Frontiers in Psychiatry. 2017;8:1–12. 28824467 10.3389/fpsyt.2017.00136 PMC5543278

[15] Clarke D, Usick R, Sanderson A, Giles-Smith L, Baker J. Emergency department staff attitudes towards mental health consumers: A literature review and thematic content analysis. International Journal of Mental Health Nursing. 2014 Jun;23(3):273–84. doi: 10.1111/inm.12040 23980913

[16] Stapleton A, Wright N. The experiences of people with borderline personality disorder admitted to acute psychiatric inpatient wards: A meta-synthesis. Journal of Mental Health [Internet]. 2019;28(4):443–57. Available from: doi: 10.1080/09638237.2017.1340594 28686468

[17] Rogers B, Dunne E. A qualitative study on the use of the care programme approach with individuals with borderline personality disorder: A service user perspective. Journal of Psychological and Mental Health Services. 2013;51(10):38–45. doi: 10.3928/02793695-20130628-03 23855437

[18] Fallon P. Travelling through the system: The lived experience of people with borderline personality disorder in contact with psychiatric services. Journal of Psychiatric and Mental Health Nursing. 2003;10(4):393–401. doi: 10.1046/j.1365-2850.2003.00617.x 12887630

[19] Ogrodniczuk JS, Kealy D, Howell-Jones G. A view from the trenches: A survey of Canadian clinicians’ perspectives regarding the treatment of borderline personality disorder. Journal of Psychiatric Practice. 2009;15(6):449–53. doi: 10.1097/01.pra.0000364286.63210.db 19934719

[20] Bodner E, Cohen-Fridel S, Mashiah M, Segal M, Grinshpoon A, Fischel T, et al. The attitudes of psychiatric hospital staff toward hospitalization and treatment of patients with borderline personality disorder. BMC Psychiatry. 2015 Dec 22;15(1):2. doi: 10.1186/s12888-014-0380-y 25609479 PMC4307152

[21] Shanks C, Pfohl B, Blum N, Black DW. Can negative attitudes toward patients with borderline personality disorder be changed? The effect of attending a STEPPS workshop. Journal of Personality Disorders. 2011 Dec;25(6):806–12. doi: 10.1521/pedi.2011.25.6.806 22217226

[22] Treloar AJC. A qualitative investigation of the clinician experience of working with borderline personality disorder. New Zealand Journal of Psychology. 2009;38(2):30–4.

[23] Hong V. Borderline personality disorder in the emergency department: Good psychiatric management. Harvard Review of Psychiatry. 2016;24(5):357–66. doi: 10.1097/HRP.0000000000000112 27603743

[24] Brodsky BS, Groves SA, Oquendo MA, Mann JJ, Stanley B. Interpersonal precipitants and suicide attempts in borderline personality disorder. Suicide and Life-Threatening Behavior. 2006;36(3):313–22. doi: 10.1521/suli.2006.36.3.313 16805659

[25] Bedics JD, Atkins DC, Harned MS, Linehan MM. The therapeutic alliance as a predictor of outcome in dialectical behavior therapy versus nonbehavioral psychotherapy by experts for borderline personality disorder. Psychotherapy. 2015;52(1):67–77. doi: 10.1037/a0038457 25751116

[26] Kverme B, Natvik E, Veseth M, Moltu C. Moving toward connectedness—A qualitative study of recovery processes for people with borderline personality disorder. Frontiers in Psychology. 2019;10:1–11. 30873097 10.3389/fpsyg.2019.00430 PMC6403141

[27] Wnuk S, McMain S, Links PS, Habinski L, Murray J, Guimond T. Factors related to dropout from treatment in two outpatient treatments for borderline personality disorder. Journal of personality disorders. 2013;27(6):716–26. doi: 10.1521/pedi_2013_27_106 23718760

[28] Kaselionyte J, Conneely M, Giacco D. “It’s a matter of building bridges. . .”—Feasibility of a carer involvement intervention for inpatients with severe mental illness. BMC Psychiatry. 2019 Sep 3;19(1). 10.1186/s12888-019-2257-6 PMC6721093 31481057

[29] Goodwin V, Happell B. Consumer and carer participation in mental health care: The carer’s perspective: Part 2—Barriers to effective and genuine participation. Issues in Mental Health Nursing. 2007 Jan 9;28(6):625–38. doi: 10.1080/01612840701354612 17613160

[30] Goodwin V, Happell B. Consumer and carer participation in mental health care: The carer’s perspective: Part 1—The importance of respect and collaboration. Issues in Mental Health Nursing. 2007 Jan 9;28(6):607–23. doi: 10.1080/01612840701354596 17613159

[31] Jones IR, Ahmed N, Catty J, McLaren S, Rose D, Wykes T, et al. Illness careers and continuity of care in mental health services: A qualitative study of service users and carers. Social Science & Medicine. 2009 Aug;69(4):632–9. doi: 10.1016/j.socscimed.2009.06.015 19577834

[32] Ng FYY, Bourke ME, Grenyer BFS. Recovery from borderline personality disorder: A systematic review of the perspectives of consumers, clinicians, family and carers. PLoS ONE. 2016;11(8):1–22. doi: 10.1371/journal.pone.0160515 27504634 PMC4978398

[33] Dunne E, Rogers B. “It’s us that have to deal with it seven days a week”: Carers and borderline personality disorder. Community Mental Health Journal. 2013;49(6):643–8. doi: 10.1007/s10597-012-9556-4 23054157

[34] Davison GC. Stepped care: Doing more with less? Journal of Consulting and Clinical Psychology. 2000 Aug;68(4):580–5. 10965633

[35] Richards DA. Stepped care: A method to deliver increased access to psychological therapies. The Canadian Journal of Psychiatry. 2012 Apr 1;57(4):210–5. doi: 10.1177/070674371205700403 22480585

[36] Paris J. Stepped care: An alternative to routine extended treatment for patients with borderline personality disorder. Psychiatric Services. 2013;64(10):1035–7. doi: 10.1176/appi.ps.201200451 23945913

[37] Gunderson JG, Links PS. Handbook of good psychiatric management for borderline personality disorder. First. Washington, DC: American Psychiatric Association Publishing; 2014.

[38] Dunston R, Lee A, Boud D, Brodie P, Chiarella M. Co-production and health system reform: From re-imagining to re-making. Australian Journal of Public Administration. 2009 Mar;68(1):39–52.

[39] McMullin C, Needham C. Co-production in healthcare. In: Brandsen T, Steen TPS, editors. Co-Production and Co-Creation: Engaging Citizens in Public Services. New York: Routledge; 2018. p. 151–9.

[40] Troup J., Lever Taylor B., Sheridan Rains L., Broeckelmann E., Russell J., Jeynes T., et al. (2022) Clinician perspectives on what constitutes good practice in community services for people with complex emotional needs: A qualitative thematic meta-synthesis. PLoS ONE 17(5). doi: 10.1371/journal.pone.0267787 35511900 PMC9070883

[41] Markkanen S, Burgess G. Introduction to co-production in services: Summary report [Internet]. 2016. Available from: https://www.researchgate.net/publication/299603822_Introduction_to_co-production_in_research_summary_report

[42] Barrett MS, Chua W-J, Crits-Christoph P, Gibbons MB, Thompson D. Early withdrawal from mental health treatment: Implications for psychotherapy practice. Psychotherapy: Theory, Research, Practice, Training. 2008;45(2):247–67. doi: 10.1037/0033-3204.45.2.247 19838318 PMC2762228

[43] Smith JA, Flowers P, Larkin MH. Interpretative phenomenological analysis. London: SAGE; 2009.

[44] Friesen L, Gaine G, Klaver E, Klingle K, Parmar D, Hrabok M, et al. Bridging the gap in community care for patients with borderline personality disorder: Protocol for qualitative inquiry into patient, caregiver, and clinician perspectives on service gaps and potential solutions for severe emotion dysregulation. JMIR Research Protocols. 2020 Aug 20;9(8):e14885. doi: 10.2196/14885 32815818 PMC7471890

[45] Eisner EW. On the art and science of qualitative research in psychology. In: Camic PM, Rhodes JE, Yardley L, editors. Qualitative research in psychology: Expanding perspectives in methodology and design. Washington: American Psychological Association; 2003. p. 17–29.

[46] World Health Organization. Facing the future: Opportunities and challenges for 21st-century public health in implementing the Sustainable Development Goals and the Health 2020 policy framework. Geneva, Switzerland; 2018.

[47] Ellis J. Researching children’s experience hermeneutically and holistically. The Alberta Journal of Educational Research. 2006;52(3):111–26.

[48] Packer MJ, Addison RB. Evaluating an interpretive account. In: Packer MJ, Addison RB, editors. Entering the circle: Hermeneutic investigation in psychology. State University of New York Press.; 1989. p. 275–92.

[49] Patterson ME, Williams DR. Collecting and analyzing qualitative data: Hermeneutic principles, methods and case examples. Advances in Tourism Applications Series. 2002;9.

[50] Bauer R, Koepke F, Sterzinger L, Spiessl H. Burden, rewards, and coping: The ups and downs of caregivers of people with mental illness. Journal of Nervous & Mental Disease. 2012 Nov;200(11):928–34. 23124175 10.1097/NMD.0b013e31827189b1

[51] Fossati A, Somma A. Improving family functioning to (hopefully) improve treatment efficacy of borderline personality disorder: An opportunity not to dismiss. Psychopathology. 2018;51(2):149–60. doi: 10.1159/000486603 29486480

[52] van Zutphen. The emotional rollercoaster called borderline personality disorder: Neural correlates of emotion regulation and impulsivity. [Maastricht]; 2017.

[53] Pigot M, Miller CE, Brockman R, Grenyer BS. Barriers and facilitators to stepped care for personality disorder in mental health services. Personality and Mental Health. 2019;13:230–8. 31411004 10.1002/pmh.1467

[54] Laporte L, Paris J, Bergevin T, Fraser R, Cardin J-F. Clinical outcomes of stepped care program for borderline personality disorder. Personality and Mental Health. 2018;12:252–64. 29709109 10.1002/pmh.1421

[55] Paris J. Stepped care for borderline personality disorder: Making treatment brief, effective, and accessible. Academic Press; 2017.

[56] Paris J. Stepped care and rehabilitation for patients recovering from borderline personality disorder. Journal of Clinical Psychology. 2015;71(8):747–52. doi: 10.1002/jclp.22202 26189972

[57] Grenyer BFS, Lewis KL, Fanaian M, Kotze B. Treatment of personality disorder using a whole of service stepped care approach: A cluster randomized controlled trial. PLoS ONE. 2018;13(11):1–13. doi: 10.1371/journal.pone.0206472 30399184 PMC6219775

[58] Huxley E, Lewis KL, Coates AD, Borg WM, Miller CE, Townsend ML, et al. Evaluation of a brief intervention within a stepped care whole of service model for personality disorder. BMC Psychiatry. 2019;19(1):1–13. 31694681 10.1186/s12888-019-2308-z PMC6836372

[59] Choi-Kain LW, Gunderson JG. Applications of good psychiatric management for borderline personality disorder: A practical guide. American Psychiatric Association Publishing; 2019.

[60] Bernanke J, McCommon B. Training in Good Psychiatric Management for borderline personality disorder in Residency: An aide to learning supportive psychotherapy for challenging-to-treat patients. Psychodynamic Psychiatry. 2018 Jun;46(2):181–200. doi: 10.1521/pdps.2018.46.2.181 29809114

[61] Substance Abuse and Mental Health Services Administration. A treatment improvement protocol: Trauma-informed care in behavioral health services. 2014. 24901203

[62] Porter C, Palmier-Claus J, Branitsky A, Mansell W, Warwick H, Varese F. Childhood adversity and borderline personality disorder: A meta-analysis. Vol. 141, Acta Psychiatrica Scandinavica. 2020. doi: 10.1111/acps.13118 31630389

[63] Solmi M, Dragioti E, Croatto G, Radua J, Borgwardt S, Carvalho AF, et al. Risk and Protective Factors for Personality Disorders: An Umbrella Review of Published Meta-Analyses of Case–Control and Cohort Studies. Frontiers in Psychiatry. 2021;12. doi: 10.3389/fpsyt.2021.679379 34552513 PMC8450571

[64] Bozzatello P, Rocca P, Bellino S. Trauma and psychopathology associated with early onset BPD: an empirical contribution. Journal of Psychiatric Research. 2020 Dec 1;131:54–9. doi: 10.1016/j.jpsychires.2020.08.038 32927365

[65] Sweeney A, Filson B, Kennedy A, Collinson L, Gillard S. A paradigm shift: Relationships in trauma-informed mental health services. BJPsych Advances. 2018 Sep;24(5):319–33. doi: 10.1192/bja.2018.29 30174829 PMC6088388

[66] Department of Health and Social Care. Our health, our care, our say: A new direction for community services [Internet]. Norwick; 2006 Jan [cited 2021 Nov 12]. Available from: https://www.gov.uk/government/publications/our-health-our-care-our-say-a-new-direction-for-community-services

[67] Voorberg WH, Bekkers VJJM, Tummers LG. A systematic review of co-creation and co-production: Embarking on the social innovation journey. Public Management Review. 2015 Oct 21;17(9):1333–57.

[68] Turakhia P, Combs B. Using principles of co-production to improve patient care and enhance value. AMA Journal of Ethics. 2017 Nov 1;19(11):1125–31. doi: 10.1001/journalofethics.2017.19.11.pfor1-1711 29168684

[69] Finamore C, Rocca F, Parker J, Blazdell J, Dale O. The impact of a co-produced personality disorder training on staff burnout, knowledge and attitudes. Mental Health Review Journal. 2020;25(3):269–80.

[70] Choi-Kain LW, Finch EF, Masland SR, Jenkins JA, Unruh BT. What works in the treatment of borderline personality disorder. Current Behavioral Neuroscience Reports. 2017;4(1):21–30. doi: 10.1007/s40473-017-0103-z 28331780 PMC5340835

[71] Rudge S, Feigenbaum JD, Fonagy P. Mechanisms of change in dialectical behaviour therapy and cognitive behaviour therapy for borderline personality disorder: A critical review of the literature. Journal of Mental Health. 2017;1–11. doi: 10.1080/09638237.2017.1322185 28480806

[72] Grenyer BFS, Bailey RC, Lewis KL, Matthias M, Garretty T, Bickerton A. A randomized controlled trial of group psychoeducation for carers of persons with borderline personality disorder. Journal of Personality Disorders. 2019;33(2):214–28. doi: 10.1521/pedi_2018_32_340 29505385

[73] Williams M, Tien H, Schaffer A, Ellis J, Cheung A, Goldstein B, et al. Hope and borderline-personality disorder as predictors of study drop-out among inpatient youth receiving psychotherapy treatment for an episode of deliberate self-harm. Biological Psychiatry. 2018 May;83(9):S155.

[74] Levy KN. The implications of attachment theory and research for understanding borderline personality disorder. Development and Psychopathology. 2005 Dec 12;17(04). doi: 10.1017/s0954579405050455 16613426

